# Mitochondrial Proteins Coded by Human Tumor Viruses

**DOI:** 10.3389/fmicb.2018.00081

**Published:** 2018-02-06

**Authors:** Ilaria Cavallari, Gloria Scattolin, Micol Silic-Benussi, Vittoria Raimondi, Donna M. D'Agostino, Vincenzo Ciminale

**Affiliations:** ^1^Veneto Institute of Oncology IOV-IRRCS, Padova, Italy; ^2^Department of Surgery, Oncology, and Gastroenterology, University of Padova, Padova, Italy; ^3^Department of Biomedical Sciences, University of Padova, Padova, Italy

**Keywords:** Mitochondria, EBV, HTLV-1, HPV, HBV, HCV, KSHV

## Abstract

Viruses must exploit the cellular biosynthetic machinery and evade cellular defense systems to complete their life cycles. Due to their crucial roles in cellular bioenergetics, apoptosis, innate immunity and redox balance, mitochondria are important functional targets of many viruses, including tumor viruses. The present review describes the interactions between mitochondria and proteins coded by the human tumor viruses human T-cell leukemia virus type 1, Epstein-Barr virus, Kaposi's sarcoma-associated herpesvirus, human hepatitis viruses B and C, and human papillomavirus, and highlights how these interactions contribute to viral replication, persistence and transformation.

## Introduction

It is estimated that about 20% of human cancer cases world-wide are caused by infection with bacteria, parasites, or viruses (de Martel et al., 2012; IARC Monograph, 2012). Among these infectious agents, viruses have the greatest reliance on cellular pathways for completion of their life cycles, and have evolved complex mechanisms to manipulate the anabolic and proliferative capacity of the host cell while minimizing effects on cell death and destruction by the immune system.

Viruses that are linked to human cancer include human-T-cell leukemia virus type 1 (HTLV-1), Epstein-Barr virus (EBV), Kaposi's sarcoma-associated herpesvirus (KSHV), hepatitis viruses B and C (HBV and HCV, respectively), high-risk genotypes of human papillomavirus (HPV, e.g., HPV-16, HPV-18), and Merkel cell polyomavirus (MCPyV). In addition, the AIDS-causing retrovirus human immunodeficiency virus is indirectly linked to cancer through its immunosuppressive effects, which favor transformation by other tumor viruses, especially EBV and KSHV (IARC Monograph, 2012).

Many of the strategies used by viruses to replicate and persist in host cells intersect at the level of mitochondria (reviewed by Claus and Liebert, 2014). This is not surprising, given the range of essential roles of these organelles in multiple cellular processes. (i) Energy supply: as the sites of pyruvate- and fatty acid oxidation, the citric acid cycle and the electron transport chain, mitochondria are the main source of ATP in most differentiated eukaryotic cells. (ii) Ca^2+^ signaling: through their ability to take up Ca^2+^ from the cytoplasm, mitochondria act as key regulators of intracellular calcium homeostasis. (iii) ROS homeostasis: the mitochondrial electron transport chain is a major source of reactive oxygen species (ROS) that influence cell turnover. (iv) Apoptosis: proteins present within mitochondria (e.g., cytochrome *c*) and on the outer mitochondrial membrane (e.g., Bax, Bak) play a key role in the triggering of intrinsic apoptosis. (v) Innate immunity: MAVS (Mitochondrial antiviral signaling protein), a component of RIG-1 (retinoic acid-inducible gene **I**) signaling, is located on the outer mitochondrial membrane.

All of the human tumor viruses except MCPyV (the most recently identified human tumor virus, associated with a rare form of skin cancer Feng et al., 2008b) code for one or more proteins that affect mitochondrial function.

The present review summarizes current knowledge on the interplay between mitochondria and proteins produced by HTLV-1, EBV, KSHV, HBV, HCV, and HPV (Table 1), and how these proteins influence the replication strategies and oncogenic properties of these viruses (Table 2).

**Table 1:** Localization and main mitochondrial effects of viral proteins.

**Virus**	**Viral Protein**	**Localization**	**Main effects on mitochondria**	**Key References**
HTLV-1	p13	IMM^a^ nucleus^f^	Fragmentation^b^ mtROS production (low doses), irreversible swelling depolarization and cytochrome *c* release (high dose)^c^ Reduced mitochondrial Ca^2+^ uptake^d^ Recruitment of SFKs to the intermembrane space^e^	Ciminale et al., 1999^b^; D'Agostino et al., 2002^a^; Silic-Benussi et al., 2009^c^; Biasiotto et al., 2010^d^; Tibaldi et al., 2011^e^; Andresen et al., 2011^f^
EBV	BHRF1	OMM^a^	Binds BH3-only proteins and inhibits Bak-Bax oligomerization in OMM^b^	Hickish et al., 1994^a^; Cross et al., 2008^b^; Flanagan and Letai, 2008^b^; Kvansakul et al., 2010^b^
KSHV	K7	Mitochondria^a^, ER^b^, nuclear membranes^c^	Blocks apoptosis by forming bridge with Bcl-2 and Caspase-3^d^	Feng et al., 2002^a^; Wang et al., 2002^a, b, c, d^
	KS-Bcl-2	Mitochondria^a^, nucleus^b^	Blocks apoptosis by binding to BH3 domains of pro-apoptotic Bcl-2 family proteins^c^ and by sequestration of Aven^d^	Kalt et al., 2010 ^a, b^; Gallo et al., 2017^a, b^; Flanagan and Letai, 2008^c^; Chau et al., 2000^d^
HBV	HBx	Nucleus^a^, cytoplasm^b^, Mitochondria^c^, in OMM^d^	Change in $\Delta \psi_{\text{m}}^{\text{e},\text{f}}$ Increased mtROS^g^ Increased COXIII activity^h^ Increased mitochondrial uptake of Ca^2+i^ Increased mitochondria fission through translocation of Drp1 and degration of Mfn2^j^ Targeting of Parkin to mitochondria and mitophagy^k^ Degradation of Mfn2^1^	Henkler et al., 2001^a, b, c^; Takada et al., 1999^c, f^; Huh and Siddiqui, 2002^d^; Rahmani et al., 2000^f^; Shirakata and Koike, 2003^f^; Clippinger and Bouchard, 2008^d, f^; Zheng et al., 2014^e, h^; Ren et al., 2016^g^;Zou et al., 2015^h^; Yang and Bouchard, 2012^i^; Kim et al., 2013a,b^j, k^
	Pol	Mitochondria^a^	n. d.	Unchwaniwala et al., 2016^a^
HCV	Core	ER^a^, OMM^b^, MAMs^c^, lipid droplets^d^	Loss of $\Delta \psi_{\text{m}}^{\text{e}}$ Increased mtROS^f^ Increased uptake of Ca^2+g^ promotion^h^ or inhibition^i^ of mitophagy	Lo et al., 1995^a^; Santolini et al., 1995^a^; Barba et al., 1997^d^; Schwer et al., 2004^b, c^; Benali-Furet et al., 2005^e, g^; Suzuki et al., 2005^a, b^; Rouille et al., 2006^a, d^; Machida et al., 2006^e^; Wang et al., 2010^b, c^;Chu et al., 2011^f^;Kim et al., 2013b^h^; Hara et al., 2014^i^
	P7	ER^a^, MAMs^b^	Loss of $\Delta \psi_{\text{m}}^{\text{c}}$	Griffin et al., 2005^b^; Haqshenas et al., 2007^a^; Qi et al., 2017^c^
	NS4A	ER, Mitochondria	Perinuclear clustering^b^ Loss of $\Delta \psi_{\text{m}}^{\text{c}}$	Nomura-Takigawa et al., 2006^a, b, c^
	NS3/4A	ER^a^, MAMs^b^, mitochondria^c^	Cleavage of MAVS^d^	Wolk et al., 2000^a, b^; Nomura-Takigawa et al., 2006^a, c^; Horner et al., 2011^d^; Bender et al., 2015^d^
HPV	E1^∧^E4	Cytokeratin network^a^, Mitochondria^b^	Dissociation from microtubules, perinuclear clustering^c^ Loss of $\Delta \psi_{\text{m}}^{\text{d}}$	Doorbar et al., 1991^a^; Raj et al., 2004^a, b, c, d^
	E2	Nucleus^a^, cytoplasm^b^, Mitochondria^c^	Perinuclear clustering^d^, loss of cristae structure^e^ Loss of $\Delta \psi_{\text{m}}^{\text{f}}$ Increased mtROS^g^	Blachon et al., 2005^a, b^; Lai et al., 2013^a, b, c, d, e, f, g^; Chen et al., 2014^f^

*ER, endoplasmic reticulum; MAM, mitochondria-associated membranes; OMM, outer mitochondrial membrane; IMM, inner mitochondrial membrane; ΔΨ_m_, mitochondrial membrane potential; mtROS, mitochondrial reactive oxygen species; cyt c, cytochrome c. Letters in superscript indicate references for each property listed columns*.

**Table 2:** Role of viral mitochondrial proteins in replication and transformation.

**Viral protein**	**Effects on virus replication**	**Role in transformation**
HTLV-1 p13	Not required for replication *in vitro* (Derse et al., 1997), but necessary for virus persistence in *in vivo* rabbit model (Hiraragi et al., 2006); mechanism unknown	Proposed negative role: interferes with transformation of fibroblasts by Myc and Ras; favors death of transformed but not normal T-cells, possibly by raising mitochondrial ROS production (reviewed by Silic-Benussi et al., 2010a)
EBV BHRF1	Not required for virus replication (Altmann and Hammerschmidt, 2005)	Required for transformation of resting B-cells, but dispensable for transformation of activated B-cells (Altmann and Hammerschmidt, 2005)
KSHV K7	Not required for virus replication (Liang et al., 2015; Gallo et al., 2017)	Possible negative role through interference with transforming activity of vGPCR (Feng et al., 2008a)
KSHV KS-Bcl-2	Required for efficient virus replication (Gelgor et al., 2015; Liang et al., 2015; Gallo et al., 2017) Not required for establishment of latent infection (Gelgor et al., 2015)	Positive role likely, through inhibition of apoptosis in infected cells
HBV HBx	Favors viral replication; enhances viral polymerase activity through Ca^2+^ signaling (Lucifora et al., 2011)	Transforms cells *in vitro* and causes tumors in transgenic mice (reviewed by Levrero and Zucman-Rossi, 2016)
HBV Pol	Needed for packaging of pregenomic RNA and reverse transcription into dsDNA genome (Bartenschlager and Schaller, 1992)	Not defined
HCV Core	Forms virion capsid (reviewed by Scheel and Rice, 2013)	Transforms cells *in vitro* and causes tumors in transgenic mice (reviewed by Banerjee et al., 2010)
HCV p7	Viroporin required for virion assembly and release (reviewed by Madan and Bartenschlager, 2015)	Not defined
HCV NS3/4A	Required for viral RNA replication, polyprotein processing and virion assembly (reviewed by Morikawa et al., 2011); cleavage of MAVS proposed to favor immune evasion (Horner et al., 2011)	Not defined; NS3 alone transforms cells *in vitro* (Sakamuro et al., 1995)
NS4A	Forms complex with NS3 (see above)	Not defined
HPV E1^∧^E4	May promote virion release through perturbation of the cytokeratin network (Raj et al., 2004)	Not defined
HPV E2	Essential for coordination of late events of viral replication (reviewed by Graham, 2016)	Negative role, through inhibition of E6 and E7 expression (reviewed by Woodman et al., 2007)

## HTLV-1

HTLV-1 is a retrovirus that infects at least 10 million people worldwide, with most cases identified in southwestern Japan, the Caribbean basin, sub-Saharan Africa, and Brazil (reviewed by Gessain and Cassar, 2012). HTLV-1 is transmitted through transfer of infected cells during breast-feeding, sexual contact, and exposure to blood and results in persistent infection, mainly in CD4+ T-cells. About 3–5% of infected patients develop an aggressive neoplasm of mature CD4+ T-cells named adult T-cell leukemia/lymphoma (ATLL) or a neurological disease named tropical spastic paraparesis/HTLV-associated myelopathy (TSP/HAM) after a latency period of decades (ATLL) or years (TSP/HAM). Other diseases associated with HTLV-1 infection include uveitis, infective dermatitis, myositis and other pathologies with an important inflammatory component (reviewed by Goncalves et al., 2010). Almost 40 years after the discovery of HTLV-1, we still lack an accurate measure of the global burden of infection, there is no HTLV-1 vaccine, and biomarkers to predict clinical outcome remain to be identified (reviewed by Willems et al., 2017).

In addition to the gag, pol, pro and env gene products produced by all retroviruses, the genome of HTLV-1 codes for nonstructural proteins named Tax, Rex, p12, p13, p21Rex, p30/Tof, and HBZ (reviewed by Journo et al., 2009; Lairmore et al., 2012). Tax and Rex are essential for completion of the replication cycle, with Tax driving transcription from the viral 5′-LTR (long terminal repeat) promoter, and Rex promoting expression of incompletely spliced transcripts, including those coding for the virion proteins. Tax and HBZ are considered to be the principal viral factors that drive development of ATLL (reviewed by Panfil et al., 2016).

### p13

p13 is an 87-amino acid protein that accumulates primarily in the inner mitochondrial membrane (Table 1; D'Agostino et al., 2002). p13 is considered to be an accessory protein, as its deletion from the viral genome does not abolish viral replication *in vitro* (Derse et al., 1997). However, studies carried out in a rabbit model of HTLV-1 infection indicated that p13 is important for establishing a persistent infection *in vivo* (Hiraragi et al., 2006).

Studies of transfected HeLa cells demonstrated that mitochondrial accumulation of p13 is directed by a 10-residue mitochondrial targeting signal near its amino terminus (MTS; amino acids 22-31) which, unlike most MTS, is not cleaved during mitochondrial import (Ciminale et al., 1999). The MTS contains 4 arginines and folds into an amphipathic α-helix (D'Agostino et al., 2002). The carboxy-terminal half of p13 contains a putative hinge (amino acids 42-48) and β-sheet (amino acids 65-75) (Silic-Benussi et al., 2010a) and a cluster of prolines that mediate binding to proteins containing SH3 domains (Ghorbel et al., 2006; Tibaldi et al., 2011).

Expression of p13 in HeLa cells alters mitochondrial morphology and produces isolated clusters of round-shaped, fragmented mitochondria (Ciminale et al., 1999). These effects depend on the presence of the 4 arginines that constitute the charged face of the amphipathic α-helix (Silic-Benussi et al., 2004). When added to isolated rat liver mitochondria, synthetic p13 protein induces an inward K^+^ current, mitochondrial swelling and loss of mitochondrial membrane potential (ΔΨ_m_). However, this depolarization triggers a compensatory increase in electron transport chain activity, which restores ΔΨ_m_ but raises levels of mitochondrial reactive oxygen species (ROS) and lowers the threshold for opening of the permeability transition pore (PTP), a channel that regulates apoptosis (reviewed by Bernardi et al., 2015). These effects are dose-dependent, as low concentrations of p13 induce mitochondrial swelling and increased mitochondrial ROS, but not mitochondrial depolarization, while high levels of p13 cause irreversible swelling, depolarization and cytochrome *c* release (Silic-Benussi et al., 2009).

p13 corresponds to the carboxy-terminal portion of p30/Tof, a nucleolar/nuclear accessory protein whose activities include suppression of Tax and Rex expression and modulation of cellular transcription (reviewed by Anupam et al., 2013). Despite this sequence overlap, p13 and p30/Tof are expressed from distinct alternatively spliced mRNAs. Studies of the temporal regulation of HTLV-1 gene expression in infected cells demonstrated that the mRNA coding for p13 is expressed as a late gene (Cavallari et al., 2011, 2013; Rende et al., 2011) and is Rex-dependent (Cavallari et al., 2015).

The structural and functional properties of p13 suggest that it may act as a viroporin (reviewed by D'Agostino et al., 2005a,b). The viroporins are a family of small, hydrophobic viral proteins with one or more membrane-spanning domains that, upon oligomerization in membranes, alter membrane permeability to ions and small molecules through the formation of channels or pores, and change the trafficking, processing and lifespan of membrane-associated proteins. Although these alterations have diverse effects on infected cells, the principal role of viroporins is to promote the assembly and egress of virus particles (reviewed by Nieva et al., 2012; Scott and Griffin, 2015).

The activity of Ca^2+^ as a second messenger is crucial for the metabolism and function of all cells, including T-cells (reviewed by Fracchia et al., 2013), the main targets of HTLV-1 infection *in vivo*. Ca^2+^ messages are delivered in the form of transient elevations in cytosolic Ca^2+^ concentration upon its release from organelles, especially the ER, and upon entry from the extracellular environment; mitochondria participate in this process through their ability to take up Ca^2+^. Experiments carried out in HeLa cells expressing organelle-targeted aequorins revealed that p13 specifically reduces mitochondrial Ca^2+^ uptake (Biasiotto et al., 2010).

The impact of p13 on mitochondrial Ca^2+^ flux might intersect with that of p12, a 99-residue viral protein that accumulates in the endoplasmic reticulum (ER) and cis-Golgi (reviewed by Van Prooyen et al., 2010). p12 increases Ca^2+^ release from the ER, thus promoting activation of NFAT (nuclear factor of activated T-cells), a key mitogenic transcription factor whose activity is controlled by the calcium-dependent phosphatase calcineurin (Kim et al., 2003). p12-mediated release of ER calcium stores and p13-mediated interference with mitochondrial calcium uptake could be predicted to amplify Ca^2+^ signals in response to T-cell receptor activation with consequent enhancement of NFAT activation (Silic-Benussi et al., 2010a). In alternative, this combination of effects could convert a Ca^2+^ signal to a prolonged increase in cytosolic Ca^2+^, which might trigger apoptosis.

Efforts to understand the function of p13 through identification of its binding partners indicated that it interacts with farnesyl pyrophosphate synthetase (FPPS) (Lefebvre et al., 2002); with members of the Src family of protein kinases (Tibaldi et al., 2011), and with the HTLV-1 regulatory protein Tax (Andresen et al., 2011). Yeast 2-hybrid screening assays also indicated binding of p13 to a protein of the nucleoside monophosphate kinase superfamily and to actin-binding protein 280 (Hou et al., 2000), but these interactions were not explored in detail.

Binding of p13 to Src family kinases (SFKs) involves p13's proline-rich C-terminal domain and the kinases' SH3 domains (Tibaldi et al., 2011). This interaction promotes the accumulation of SFKs in the mitochondrial intermembrane space and enhances their tyrosine kinase activity, while interfering with targeting of p13 to the inner membrane and attenuating the effects of p13 on ΔΨ_m_ (Tibaldi et al., 2011).

Experiments carried out in 293T cells and HeLa cells showed that when co-expressed with Tax, p13 undergoes ubiquitination, becomes more stable, and is partially rerouted to nuclear speckles containing Tax (Andresen et al., 2011). These effects are mediated by direct interaction of Tax with p13 through a disulphide bond involving cysteine 27 in p13. Results of co-immunoprecipitation and LTR-reporter assays showed that the p13-Tax interaction interferes with binding of Tax to the transcription cofactor CBP/p300 and leads to a decrease in Tax-mediated viral gene transcription, a crucial step in viral replication. The finding that substitution of the p13 start codon with isoleucine in an HTLV-1 proviral clone resulted in an increase in viral gene expression supports the proposal that p13 might acts as a negative regulator of HTLV-1 replication (Andresen et al., 2011).

The effect of ablation of p13 expression on the ability of the virus to transform primary T-cells needs to be investigated. However, results of studies of primary cells and cell lines forced to express p13 indicate that it may limit the oncogenic potential of HTLV-1. p13 interfered with the ability of Myc and Ras to transform rat embryo fibroblasts, and HeLa cells expressing p13 exhibited a proliferation defect *in vitro* and were less tumorigenic in nude mice compared to parental cells (Silic-Benussi et al., 2004). Subsequent comparisons of the effects of p13 in the T-cell line Jurkat and normal primary T-cells showed that the protein induced ROS production in both cell types, and slowed proliferation and increased apoptotic death in Jurkat cells, but activated normal primary T-cells (Silic-Benussi et al., 2010a,b). These observations suggest that p13 might expand the pool of untransformed infected cells and on the other hand favor the elimination of transformed cells, thus increasing HTLV-1's adaptation to the host and lifelong persistence of the infection. It will be interesting to determine whether the effects of p13 on T-cell activation are connected to its impact on Ca^2+^ homeostasis.

## EBV and KSHV

EBV and KSHV are members of the γ-herpesvirus subfamily of the Herpesviridae, a family of large, enveloped viruses with a linear double-stranded DNA genome. EBV targets mainly B-cells and epithelial cells, while KSHV targets mainly B-cells and endothelial cells. Like all herpesviruses, the life cycle of EBV and KSHV comprises latent states that favor viral persistence and a lytic phase that produces virus particles. EBV infection is present in more than 90% of adults in a uniform distribution worldwide. Although studies of global KSHV epidemiology are incomplete, it appears to be more restricted geographically to parts of sub-Saharan Africa, the Mediterranean basin, Brazil and China, and in certain subpopulations such as men who have sex with men (Morrison et al., 2015). Primary infection with EBV and KSHV occurs mainly through contact with saliva during early childhood and usually does not cause overt clinical symptoms, except for a self-limiting polyclonal B-lymphoproliferative disease termed infectious mononucleosis that arises in at least 25% of patients who become infected with EBV in adolescence or early adulthood. Neoplasias associated with EBV include an endemic form of Burkitt's lymphoma, post-transplant lymphoproliferative disorders, some cases of Hodgkin's lymphoma, and nasopharyngeal carcinoma (reviewed by Young et al., 2016). KSHV is linked to 3 types of tumors, with immunosuppression representing a risk factor: the endothelial-derived tumor Kaposi's sarcoma (KS, always KSHV-positive), a B-cell malignancy named primary effusion lymphoma (PEL; always KSHV-positive, sometimes also EBV-positive) and a plasmablastic form of the B-lymphoproliferative disorder multicentric Castleman disease (MCD, KSHV-positive in about half of cases) (reviewed by Bhutani et al., 2015). EBV and KSHV share 59 gene homologs whose similarity ranges from 41 to 75% (Russo et al., 1996); both viruses possess genes that closely resemble cellular genes. Proteins of EBV and KSHV with an impact on mitochondria include EBV BHRF1, BZLF1, and LMP2A, and KSHV K7 and KS-Bcl-2 (Table 1).

### EBV: BHRF1, BZLF1 BALF1, LMP2A

**Figure 1:**
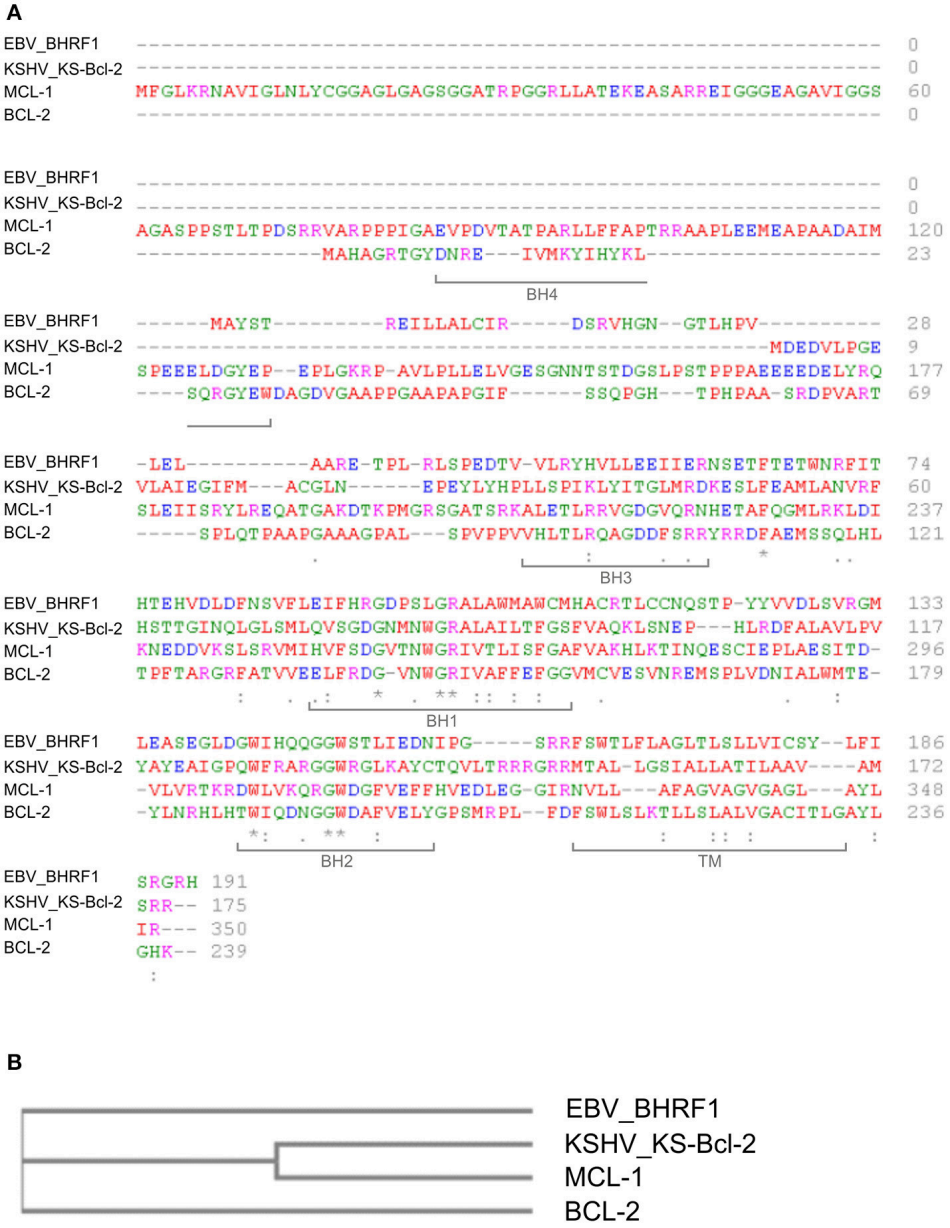
Sequence comparison of human Bcl-2 family proteins and viral homologues. **(A)** Multiple sequence alignment. Protein sequences were obtained from the UniProt database (http://www.uniprot.org/) and analyzed with the online software Clustal Omega (http://www.ebi.ac.uk/Tools/msa/clustalo/). Amino acids are labeled in different colors according to their biochemical properties (red: small/hydrophobic; blue: acidic; magenta: basic; green: hydroxyl/sulfhydryl/amine/Glycine). Asterisks indicate single conserved residues; periods indicate residues with similar properties, and brackets indicate locations of the BH and TM domains in Bcl-2 (UniProtKB–P10415 (BCL2_HUMAN). Accession IDs: KS-Bcl-2 (>sp|F5HGJ3|ARBH_HHV8P); BHRF1 (>sp|P03182|EAR_EBVB9); Bcl-2 (>sp|P10415|BCL2_HUMAN)); Mcl-1 (>sp|Q07820|MCL1_HUMAN). **(B)** Cladogram representing the similarity between human Bcl-2 and Mcl-1 and the viral orthologs of EBV (EBV_BHRF1) and KSHV (KSHV_KS-Bcl-2). The cladrogram was generated with the online software Clustal Omega.

BHRF1 is a 191-amino acid, early lytic-phase EBV protein with similarity to cellular Bcl-2 family proteins (Pearson et al., 1987) that blocks the mitochondrial arm of apoptosis mediated by pro-apoptotic Bcl-2 proteins (Henderson et al., 1993). In this manner, BHRF1 promotes the survival of EBV-infected cells, thus promoting viral persistence/replication. The production of viral Bcl-2 homologs (v-Bcl-2) is shared by several other viruses, including KSHV, which produces KS-Bcl-2 (see below; reviewed by Kvansakul et al., 2017) (Figures 1A,B).

BHRF1 accumulates in the outer mitochondrial membrane (OMM) of B-cells, a distribution similar to that of Bcl-2 (Hickish et al., 1994). Mitochondrial targeting of BHRF1 is directed by a C-terminal sequence that shares homology with the transmembrane domain (TM) present in some cellular Bcl-2 family members (Bcl-2 38%, Bcl-xl 32%, Bax 34%) (Hickish et al., 1994). As shown in Figure 1A, BHRF1 also has all of the Bcl-2 homology (BH) domains except for BH4, which is in general poorly conserved among Bcl-2 family members.

The anti-apoptotic effects of BHRF1 are mainly due to its ability to bind to BH3-only pro-apoptotic Bcl-2 proteins and to inhibit the formation of Bax/Bak oligomers in the OMM (Cross et al., 2008; Flanagan and Letai, 2008; Kvansakul et al., 2010; Milian et al., 2015). BHRF1 binds to the BH3-only proteins Bim, Bak, Bid and PUMA (Kvansakul et al., 2010). Structural studies of the BHRF1:Bim/Bak complexes indicated that an aspartic acid at position 100 of BHRF1 is essential for its association with the BH3 domains of Bim and Bak, in interactions similar to those occurring between Bim and the cellular anti-apoptotic protein Bcl-xL (Kvansakul et al., 2010). EBV also codes for BALF1, a cytoplasmic protein with homology to Bcl-2 family members that was shown by one group to interfere with the anti-apoptotic activity of BHRF1 (Bellows et al., 2002) but by others to inhibit apoptosis (Marshall et al., 1999) and promote transformation (Hsu et al., 2012).

BZLF1 (also known as ZEBRA, *Zta*) is a 245-amino acid EBV protein of the basic leucine zipper (b-zip) family that is essential for transcription of EBV lytic-phase genes and replication of viral genomes (reviewed by McKenzie and El-Guindy, 2015). Experiments carried out in EBV-positive B-cell lines indicated that BZLF1 can interact with the mitochondrial protein mtSSB, a single-stranded DNA-binding protein that is needed for replication of the mitochondrial genome. Cells in the lytic phase showed reduced mtDNA synthesis and contained fewer mitochondrial genomes, while silencing of mtSSB interfered with BZLF1-dependent entry into the lytic phase. Imaging analyses of cells expressing FLAG-tagged BZLF1 indicated that BZLF1 partially redirects mtSSB from mitochondria to the nucleus, which is the main site of BZLF1 accumulation (Wiedmer et al., 2008). Expression of BZLF1 in HeLa cells resulted in fused masses of mitochondria, some of which were localized near the nucleus (LaJeunesse et al., 2005); it is however unclear how BZLF1 induces these changes in mitochondrial morphology.

EBV LMP2A (latent membrane protein 2A) is a 497-amino acid protein with 12 transmembrane domains that accumulates mainly in plasma membrane rafts, where it influences signal transduction pathways affecting cell activation, proliferation, survival and migration (reviewed by Cen and Longnecker, 2015). Pal et al. (2014) showed that LMP2A has indirect effects on mitochondria. Expression of LMP2A in EBV-negative gastric- and breast cancer cell lines resulted in increased mitochondrial fission accompanied by an increase in migration and induction of the epithelial-mesenchymal transition. These effects were attributed to LMP2A's ability to stimulate the Notch pathway, which in turn upregulates Drp1 (dynamin-related protein 1), a protein that induces mitochondrial fission (Pal et al., 2014).

### KSHV: K7, KS-Bcl-2

K7 is a 126-amino acid protein that shows homology to the cellular protein Survivin, a member of the family of inhibitor of apoptosis proteins (IAPs). K7 is detected in the endoplasmic reticulum, nucleus and in mitochondria (Feng et al., 2002; Wang et al., 2002) and is expressed early after induction of the lytic cycle (Wang et al., 2002). It contains an amino-terminal, atypical MTS, a putative transmembrane domain partially overlapping the MTS, a baculovirus IAP repeat (BIR), and a carboxy-terminal BH2 domain (Wang et al., 2002).

K7's BH2 and BIR domains mediate its direct interaction with Bcl-2 and activated caspase-3, respectively; the resulting bridge between K7, Bcl-2, and activated caspase-3 inhibits the apoptotic caspase cascade (Wang et al., 2002). Another anti-apoptotic function of K7 involves its association with CAML (calcium-modulating cyclophilin ligand), an ER protein that controls intracellular Ca^2+^ homeostasis (Feng et al., 2002). The CAML-K7 interaction alters the changes in cytosolic Ca^2+^ induced by thapsigargin, an inhibitor of the sarcoplasmic/endoplasmic Ca^2+^-ATPase (SERCA), whose role is to transport Ca^2+^ from the cytosol to the ER. In this manner, K7 protects thapsigargin-treated cells from Ca^2+^-induced loss of ΔΨ_m_ and apoptosis (Feng et al., 2002).

K7 also interacts with a helix-coiled region (HC) of Rubicon, an inhibitor of autophagosomal maturation that forms a complex with Beclin 1, UVRAG and Vps34 (Liang et al., 2013). K7 increases the expression of Rubicon and enhances its interaction with the autophagy machinery, resulting in a block in the autophagy process. Cells expressing a virus knocked out for K7 were able to produce virus, but expression of the viral proteins K3 and K8 was reduced (Liang et al., 2013). K7-mediated regulation of K3 and K8 expression would have an important effect on viral persistence, as K3 induces internalization and degradation of MHC-I complexes, thus favoring the escape of virus-infected cells from the control of the immune system, while K8 is part of the viral lytic DNA replication complex (Liang et al., 2013).

Another binding partner of K7 is vGPCR, a KSHV homolog of the human interleukin-8 receptor that induces tumors in nude mice. K7 induces the degradation of vGPCR through the ER-associated degradation pathway, resulting in a decrease in tumorigenicity (Feng et al., 2008a).

KS-Bcl-2 is a 175-amino acid protein that is expressed from KSHV ORF16. KS-Bcl-2 shares about 60% overall sequence homology with cellular proteins of the Bcl-2 family, with high conservation of the BH1, BH2 and TM domains (Figure 1A; Sarid et al., 1997). KS-Bcl-2 appears to be more closely related to MCL-1 than to Bcl-2 in terms of sequence homology (Figure 1B) and functional activity (Flanagan and Letai, 2008).

The first study of KS-Bcl-2 described a punctate cytoplasmic distribution, indicative of accumulation in organelle membranes, but did not identify these organelles (Sarid et al., 1997). More recent studies of KS-Bcl-2 indicated its partial localization in mitochondria and in the nucleus (Gallo et al., 2017) or nucleoli (Kalt et al., 2010); these studies employed tagged versions of KS-Bcl-2 and a fluorescent marker to distinguish the mitochondrial compartment in different cell lines. The combination of tagging and cell lines used in these studies likely influenced KS-Bcl-2's localization: in the study by Gallo et al., when expressed in 293A cells, FLAG-tagged KS-Bcl-2 colocalized with the fluorescent mitochondrial marker in compact crescent-shaped structures lying against the nucleus, while in HUVEC cells the protein colocalized with the marker in structures resembling the web-like mitochondrial network (Gallo et al., 2017).

The KS-Bcl-2 mRNA is detected in KS lesions and in PEL cells upon induction of the lytic cycle and is required for lytic reactivation and replication (Gelgor et al., 2015; Liang et al., 2015). The best-defined roles of KS-Bcl-2 are to inhibit both apoptosis and autophagy. KS-Bcl-2 impedes apoptosis through interactions with the BH3 domains of pro-apoptotic proteins of the Bcl-2 family (Bim, Bid, PUMA, Bik, NOXA, Bmf) (Flanagan and Letai, 2008) and through its association with Aven, a protein that interferes with the ability of Apaf-1 to activate caspase 9 (Chau et al., 2000). Inhibition of autophagy by KS-Bcl-2 is mediated by its binding to Beclin 1, a key component of the autophagy pathway (Pattingre et al., 2005). Interestingly, the role of KS-Bcl-2 in promoting viral reactivation and replication is apparently independent from its effects on apoptosis and autophagy (Gelgor et al., 2015; Liang et al., 2015).

The nuclear targeting of KS-Bcl-2 was mapped to its amino-terminal 17 amino acids and was found to be important for the protein's effects of viral replication (Gallo et al., 2017). Nucleolar accumulation of KS-Bcl-2 depended on its association with the cellular nucleolar protein GLTSCR2/PICT-1, and interfered with KS-Bcl-2's anti-apoptotic properties (Kalt et al., 2010).

## HBV

HBV is a small, enveloped DNA virus of the Hepadnaviridae family that is transmitted parenterally through blood and other body fluids. Most HBV-infected patients are asymptomatic or present signs of acute liver disease and inflammation. Active viral replication and insufficient immune clearance may result in chronic hepatitis, with tissue injury, inflammation and regeneration that can progress to cirrhosis, liver failure and hepatocellular carcinoma (HCC), an aggressive neoplasm with a dismal prognosis. Despite the availability of a prophylactic vaccine since the 1980s, approximately 257 million persons are chronically infected with HBV worldwide, with high-prevalence areas in the African and Western Pacific regions (statistics for 2015, World Health Organization, 2017).

HBV produces two proteins that interact with mitochondria: HBx and Pol (Table 1).

### HBx

The 154-amino acid HBx protein favors HBV replication, influences cellular transcription, signal transduction, cell proliferation and survival, and is considered to be a key contributor to the oncogenic potential of HBV (reviewed by Benhenda et al., 2009; Motavaf et al., 2013). Takada et al. provided the first evidence for mitochondrial accumulation of HBx, which was accompanied by aggregation of mitochondria near the nucleus, accumulation of p53 in mitochondria, loss of ΔΨ_m_ and increased apoptotic death (Takada et al., 1999). A subsequent study revised HBx's targeting properties to include the nucleus and cytoplasm (Henkler et al., 2001). The mitochondrially-associated fraction of HBx was detected in the outer mitochondrial membrane in the hepatoma cell line Huh7, in the hepatocarcinoma cell line HepG2, and in primary rat hepatocytes (Huh and Siddiqui, 2002; Clippinger and Bouchard, 2008). Experiments carried out with GFP fusion proteins in Huh7 cells indicated that residues 68-117 of HBx confer mitochondrial targeting (Shirakata and Koike, 2003).

HBx was shown to induce loss of ΔΨ_m_ when expressed in Huh7 cells (Takada et al., 1999; Rahmani et al., 2000; Shirakata and Koike, 2003) or HepG2 cells (Clippinger and Bouchard, 2008). However, primary rat hepatocytes expressing HBx were protected from loss of ΔΨ_m_ induced by treatment with TNF-α, a property attributed to HBx's ability to induce the NF-κB pathway (Clippinger and Bouchard, 2008). More recently, Zheng et al. (2014) showed that HBx increases ΔΨ_m_ in HepG2 cells, an effect that was correlated with increased levels of cytochrome *c* oxidase III (COXIII) and increased COX activity. HBx-induced upregulation of COXIII expression and activity were also observed in the immortalized hepatocyte cell line HL-7702 (Zou et al., 2015). Although no changes in ΔΨ_m_ were observed in this system, HBx increased cellular ROS production (Zou et al., 2015).

An HBx-mediated increase in cellular ROS was associated with activation of the STAT3 and NF-κB pathways (Waris et al., 2001), both of which are important in inflammation and transformation. HBx-induced cellular ROS was also accompanied by induction of cyclooxygenase 2 (COX-2), an enzyme that catalyzes the production of mediators of the inflammatory response (Lim et al., 2010). The observation that ROS-enhancing agents such as the chemotherapeutic drug adriamycin increase the stability of HBx protein suggests the existence of a ROS-HBx positive feedback loop (Wang et al., 2003). Such a positive role for ROS would fit in with results of a recent study which indicated that HBV infection induces oxidative stress, in part through the ability of HBx to increase mitochondrial ROS, and that ROS favor replication of the virus (Ren et al., 2016).

Binding partners of HBx in mitochondria include the voltage-dependent anion channel 3 (VDAC3), which resides in the OMM (Rahmani et al., 2000), and heat shock protein 60 (HSP60), a multifunctional chaperone that is located mainly in the mitochondrial matrix (Tanaka et al., 2004). Studies carried out in Huh7 cells showed that HBx-induced ROS promotes the translocation of the serine/threonine kinase Raf-1 (C-Raf) from the cytoplasm to mitochondria, where it forms complexes with HBx (Chen and Siddiqui, 2007).

HBx-induced alterations in intracellular calcium signaling play an important role in HBV replication (Bouchard et al., 2001). HBx increases the basal levels of cytosolic Ca^2+^ in HepG2 cells (McClain et al., 2007) and augments the spike in cytosolic Ca^2+^ levels provoked by ATP (Chami et al., 2003). The HBx-mediated increase in cytosolic Ca^2+^ reflects influx of Ca^2+^ into cells due to store-operated calcium entry and is associated to greater uptake of Ca^2+^ by mitochondria (Yang and Bouchard, 2012).

HBx-expressing cells also show changes in mitochondrial dynamics with increased fission, which results both from phosphorylation and mitochondrial translocation of the fission protein Drp1 and from ubiquitination/degradation of Mitofusin 2 (Mfn2), a protein that mediates mitochondrial fusion (Kim et al., 2013a). The study by Kim et al. also showed that HBx promotes mitophagy, a specialized form of autophagy that eliminates dysfunctional mitochondria. This effect was attributed to increased expression and mitochondrial targeting of Parkin, a ubiquitin ligase whose substrates provide a signal for sequestration and degradation of damaged mitochondria (reviewed by Bernardini et al., 2017). Thus, by enhancing mitophagy, HBV could promote cell survival and possibly viral persistence. On the other hand, induction of mitochondrial fission may lead to mitochondrial injury, which might play a role in the pathogenesis of HBV-related liver disease (Kim et al., 2013a).

HBx can either directly affect apoptosis or modify the response of cells to apoptotic stimuli such as TNF-α (reviewed by Rawat et al., 2012). The varied effects of HBx on cell death likely depend on its expression levels and on the cell context.

### Pol

HBV Pol is an 832-residue protein whose principal role is to direct packaging of viral pregenomic RNA (pgRNA) molecules into capsids and to reverse-transcribe the RNA into the dsDNA genome. In a study of the sites of HBV replication, Pol was observed to accumulate mainly in mitochondria (Unchwaniwala et al., 2016). A segment of Pol spanning residues 141-160 functioned as a mitochondrial targeting sequence (MTS) when attached to GFP and was important for pgRNA packaging, but could be deleted from Pol without abolishing its mitochondrial targeting, indicating that additional amino-terminal sequences contribute MTS properties. Based on the finding that neither the pgRNA nor the viral Core protein (Cp) localized to the mitochondria during replication, Unchwaniwala et al. (2016) suggested that the binding of pgRNA to the MTS may block mitochondrial localization, so that Pol bound to pgRNA would be incorporated into in capsids, while unbound Pol would accumulate in mitochondria. Although the role of Pol in mitochondria remains to be understood, the protein is known to have diverse activities besides HBV genome packaging and reverse transcription, including interference with the antiviral interferon response (Wu et al., 2007; Wang and Ryu, 2010), an effect that would favor viral persistence in chronically infected patients.

## HCV

HCV belongs to the *Flaviviridae* family of small, enveloped viruses with a single-stranded RNA genome. About 80–85% of HCV-infected persons fail to eliminate the virus due to the ability of HCV to evade innate and adaptive immune surveillance. Persistent HCV infection is associated with liver pathologies (chronic hepatitis, hepatic steatosis, hepatic fibrosis, cirrhosis, and hepatocellular carcinoma) and lymphoproliferative disorders. Although new treatment regimens with direct-acting antivirals (DAAs) are capable of eliminating the infection, HCV remains an important clinical and social problem due to the high cost of DAAs, susceptibility of cured patients to reinfection, and the lack of a prophylactic vaccine (reviewed by Webster et al., 2015; Dustin et al., 2016). Approximately 71 million persons are chronically infected with HCV, in a heterogeneous world-wide distribution with higher prevalence in the Eastern Mediterranean and European regions (statistics for 2015, World Health Organization, 2017).

The 9.6 kb HCV genome codes for a polyprotein precursor that is cleaved by viral and cellular proteases into the structural proteins Core, E1 and E2, which make up the virion, and nonstructural proteins p7, NS2, NS3, NS4A, NS4B, NS5A, and NS5B, which are involved in polyprotein processing, genome replication and virion assembly (reviewed by Scheel and Rice, 2013). The viral life cycle takes place in the ER and an ER-derived membranous web associated with lipid droplets, and results in ER stress (Dash et al., 2016). Core, p7, NS3/4, NS4A, and NS5A show partial accumulation in mitochondria or MAM (Table 1).

### Core

Core is a 177-amino acid protein that is derived from the amino-terminal end of the polyprotein precursor by two proteolytic cleavage events (Okamoto et al., 2008). In addition to forming the virion capsid, Core has multiple effects on viral and cellular pathways, and can protect against or sensitize cells to apoptotic stimuli (reviewed by Kao et al., 2016). Studies of cell lines ectopically expressing Core indicated its accumulation mainly in the ER (Lo et al., 1995; Santolini et al., 1995), in both the ER and outer mitochondrial membrane (Suzuki et al., 2005), or in MAMs and the OMM (Schwer et al., 2004). The association of Core with the ER/MAMs/OMM is mediated by an amphipathic alpha helical sequence near its carboxy terminus (Schwer et al., 2004; Suzuki et al., 2005).

The targeting properties of Core are influenced by its interactions with other viral proteins. When expressed with E1, Core was targeted mainly to lipid droplets (Barba et al., 1997). Analyses of HCV-infected Huh7 cells indicated accumulation of Core mainly in lipid droplets and associated membranes (Rouille et al., 2006) or mainly in the ER and to a lesser extent in mitochondria (Schwer et al., 2004). The fraction of Core associated with mitochondria in infected cells is restricted to the outer mitochondrial membrane (OMM) or possibly MAMs (Wang et al., 2010).

The phenotype of Core-transgenic mice includes hepatic steatosis and hepatocellular carcinoma (Moriya et al., 1997, 1998). The hepatocytes of these mice showed age-dependent accumulation of Core in the nucleus and in morphologically altered, dysfunctional mitochondria (Moriya et al., 1998), and signs of oxidative stress (Moriya et al., 2001), suggesting a direct role for Core in the oxidative stress and impaired mitochondrial function observed in patients with HCV-induced hepatitis (Farinati et al., 1995; Barbaro et al., 1999). Core was also detected in mitochondria of hepatocytes from mice co-expressing Core, E1, E2, and p7; the mitochondria contained reduced levels of glutathione and NADPH, indicating oxidative stress, and showed impaired Complex I activity, reduced respiration, and increased ROS (Korenaga et al., 2005).

Further studies of Core-expressing cell lines confirmed that it induces mitochondrial ROS (Okuda et al., 2002; Chu et al., 2011), as well as loss of ΔΨ_m_ (Benali-Furet et al., 2005; Machida et al., 2006), and release of cytochrome *c* into the cytoplasm (Okuda et al., 2002; Benali-Furet et al., 2005). Other effects of Core include increased lipid peroxidation (Okuda et al., 2002; Machida et al., 2006), accumulation of lipid droplets (Chu et al., 2011), increased autophagy (Chu et al., 2011), and apoptosis (Benali-Furet et al., 2005). Studies in Huh7 cells and the B-cell line Raji linked the effects of Core on ΔΨ_m_ and cellular ROS to activation of the STAT3 pathway and induction of DNA damage (Machida et al., 2006).

Core also induces an accumulation of Ca^2+^ in the cytoplasm due to a defect in SERCA function (Benali-Furet et al., 2005). The consequent loading of mitochondria with Ca^2+^ favors mitochondrial ROS production and triggers the mitochondrial permeability transition (Li et al., 2007). Augmented mitochondrial uptake of Ca^2+^ as well as inhibition of Complex I activity and increased ROS and glutathione oxidation are also observed when Core is added to isolated mitochondria (Korenaga et al., 2005). The effects of Core on intracellular Ca^2+^ likely contribute to the signs of ER stress observed in an osteosarcoma cell line expressing HCV (Piccoli et al., 2006, 2007, 2009). In Huh7 cells, HCV was found to favor ROS induction, loss of ΔΨ_m_ and apoptosis triggered by treatment with the pro-oxidant t-BOOH; interestingly, these effects that were lost in the absence of Core, E1, E2, and p7 (Wang et al., 2010).

Analyses of Core deletion mutants indicated that its first 75 amino acids mediate binding to the mitochondrial matrix protein HSP60, and are required for Core-mediated induction of intracellular ROS (measured using DCF-DA) and sensitization to TNF-α-induced apoptosis (Kang et al., 2009); the location in which the Core-HSP60 interaction influences ROS production was not identified.

Core also interacts with Prohibitin, a multifunctional protein that influences mitochondrial function and apoptosis (reviewed by Peng et al., 2015). Core-expressing HepG2 cells expressed increased levels of Prohibitin, but reduced levels of COX subunits I and II, suggesting that the Core-Prohibitin interaction interferes with proper assembly and activity of respiratory chain complexes, which would be expected to result in increased mitochondrial ROS production (Tsutsumi et al., 2009).

Studies of the impact of HCV on mitophagy indicated either a promoting (Kim et al., 2013b) or blocking (Hara et al., 2014) effect. The inhibitory effect was attributed to the ability of Core to interact with and inhibit mitochondrial accumulation of Parkin (Hara et al., 2014). Persistence of damaged mitochondria due to the Core-Parkin interaction may thus contribute to HCV-associated liver damage and HCC.

Alcohol consumption worsens the clinical course of chronic hepatitis C. A study of Huh7 cells provided evidence that Core may contribute to oxidative stress that arises during the metabolism of ethanol by cytochrome p4502E1 (CYP2E1) (Otani et al., 2005).

Hepatitis C patients and Core-transgenic mice show hepatic accumulation of iron (Farinati et al., 1995; Moriya et al., 2010). In iron-overload experiments, hepatocytes from Core-transgenic mice and Core-expressing HepG2 cells showed impaired upregulation of the antioxidant heme-oxygenase-1, which is normally induced by excess iron (Moriya et al., 2010). Such a defect in the iron-overload response could contribute to deregulated ROS production and scavenging (Fujinaga et al., 2011).

### p7, NS3/4A, and NS5A

There is also evidence that the HCV nonstructural proteins p7, NS3/4A, and NS5A influence mitochondrial function. p7 is a 63-amino acid hydrophobic protein that forms hexameric ion channels in membranes and is classified as a viroporin (reviewed by Nieva et al., 2012). p7 is required for the assembly and release of infectious virions, thus making it a potential target for antiviral therapy (reviewed by Madan and Bartenschlager, 2015). p7 accumulates mainly in the ER (Carrere-Kremer et al., 2002; Isherwood and Patel, 2005; Haqshenas et al., 2007; Vieyres et al., 2013) and in MAMs (Griffin et al., 2004, 2005). A recent study of the impact of HCV on the interferon response indicated that p7 induces mitochondrial membrane depolarization and binds to IFI6-16, an interferon response protein that stabilizes ΔΨ_m_, an effect that is counteracted by p7 (Qi et al., 2017).

NS3 is a 631-amino acid protein with serine protease- and RNA helicase activities that, in association with the 54-amino acid accessory protein NS4A, forms a membrane-bound complex that is required for HCV RNA replication, polyprotein processing and virion assembly (reviewed by Morikawa et al., 2011), and is a target of DAAs. NS3/4A also cleaves several cellular proteins, including MAVS, a component of the innate immune response signaling pathway triggered by binding of pathogen-associated molecular patterns (PAMPs) to RIG-1 (Meylan et al., 2005; Belgnaoui et al., 2011). In Huh7 cells, MAVS is detected in peroxisomes, mitochondria and MAMs (Seth et al., 2005; Horner et al., 2011) and is susceptible to cleavage by NS3/4A in these compartments (Horner et al., 2011; Bender et al., 2015). Consistent with these observations, there is evidence that NS3/4A is partially targeted to mitochondria (Wolk et al., 2000; Nomura-Takigawa et al., 2006). Huh7 cells expressing both NS3 and NS4A showed increased sensitivity to an apoptosis-inducing drug, suggesting that NS3/4A may contribute to HCV-associated hepatic injury by priming cells to death stimuli (Nomura-Takigawa et al., 2006). Huh7 cells expressing NS4A alone showed accumulation of the protein in the ER and in mitochondria that were perinuclear, doughnut-shaped, and depolarized. NS4A-expressing cells released cytochrome *c* in the cytoplasm and showed increased spontaneous death compared to control cells or cells coexpressing NS3 and NS4A (Nomura-Takigawa et al., 2006).

NS5A is a 467-amino acid ER/membranous web-associated phosphoprotein that regulates viral replication and assembly, and is a target of DAA (reviewed by Ross-Thriepland and Harris, 2015). When expressed in Huh7 cells, NS5A activates the NF-κB and STAT3 pathways through Ca^2+^-mediated induction of oxidative stress (Gong et al., 2001). Studies of a GFP-NS5A fusion protein in HEK293 cells indicated a role for NS5A in tethering the ER to mitochondria through a mechanism that involves binding of NS5A to phosphatidylinositol 4-kinase PI4KA III-α (PI4KA) (Siu et al., 2016). GFP-NS5A-expressing cells contained fragmented mitochondria but were more resistant to apoptosis induced by hydrogen peroxide compared to control cells, suggesting that NS5A may favor survival of HCV-infected cells under conditions of oxidative stress (Siu et al., 2016).

## HPV

Human papillomaviruses (HPVs), members of the Papillomaviridae family, are small non-enveloped viruses that possess a double-stranded circular DNA genome and show a tropism for epithelial cells (reviewed by Doorbar et al., 2015). The HPV genome codes for 6 early genes named E1, E2, E4, E5, E6, and E7, which regulate virus replication, and 2 late genes named L1 and L2, which make up the virion capsid. The pattern of HPV gene expression is tightly controlled by the differentiation status of the infected cell: after penetration into the mucosal or cutaneous epithelium through microlesions, replication initiates with expression of early genes in the basal layer, while expression of late proteins making up the virus particles is restricted to the upper layers (Tomaic, 2016).

HPV is classified into more than 200 genotypes based on the sequence of the L1 (major capsid) gene. The genotypes are grouped into 5 genera, the 2 most numerous of which are named alpha and beta. Alpha-HPVs are further classified as low-risk or high-risk based on their connection with cancer (reviewed by Egawa et al., 2015). The low-risk viruses (e.g., HPV-6 and HPV-11) cause warts and other benign proliferative lesions of the skin and mucosa, while high-risk viruses (e.g., HPV-16 and HPV-18) are associated with cervical carcinoma and cancers of the anogenital and head-and neck regions. Beta-HPVs infect cutaneous epithelia and may contribute to the initiation of non-melanoma skin cancers, with immunosuppression representing a risk factor (Tomaic, 2016). HPV-mediated transformation is driven by the viral proteins E6 and E7 (reviewed by Doorbar et al., 2015; Tomaic, 2016). The frequency of HPV-associated pathologies should be reduced with the availability, since 2006, of vaccines based on virus-like particles containing the L1 protein of selected high-risk or high- plus low-risk genotypes (reviewed by Harper and DeMars, 2017).

### E1^∧^E4

**Figure 2:**
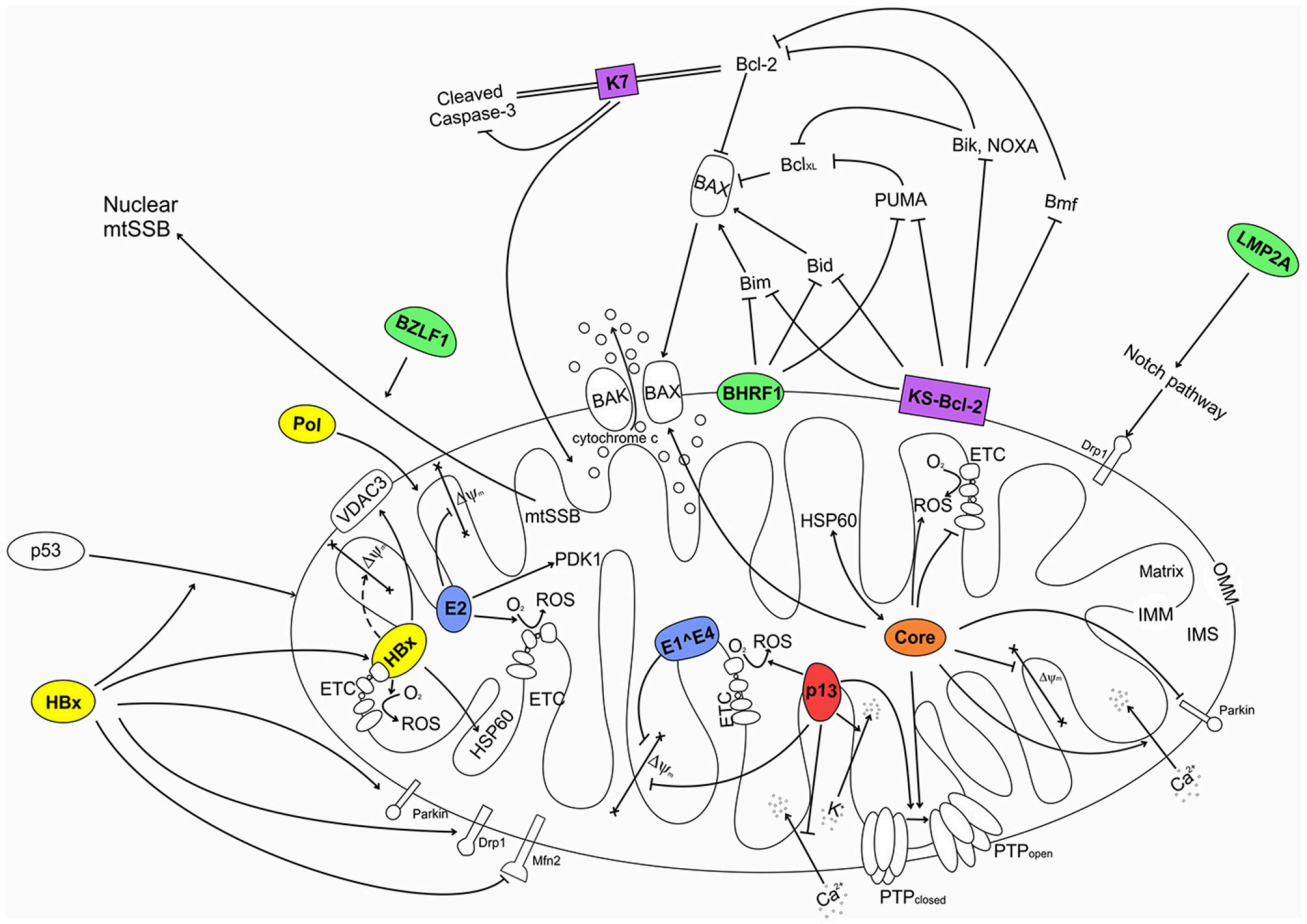
Interactions of human tumor virus proteins with mitochondria. HTLV-1 (red): p13 causes an inward K^+^ current that leads to mitochondrial swelling, depolarization and increased ROS production that lowers the PTP opening threshold. p13 also reduces mitochondrial Ca^2+^ uptake. EBV (green): The Bcl-2 homolog BHRF1 localizes at the OMM and binds to Bim, Bid and PUMA, resulting in the inhibition of Bax translocation to the OMM. BZLF1 interacts with mtSSB. LMP2A increases expression of Drp1 (dynamin-related protein 1) through stimulation of the Notch pathway. KSHV (purple): The Mcl-1 homolog KS-Bcl-2 localizes at the OMM and can bind and inhibit a variety of BH3-only proteins, resulting in the inhibition of Bax-Bak oligomerization at the OMM. The K7 protein forms a bridge between cellular Bcl-2 and cleaved Caspase-3, resulting in inhibition of Caspase-3 activity. HBV (yellow): HBx interacts with the Complex IV subunit COXIII and increases ROS generation by the ETC; HBx can interact with VDAC3 and HSP60. Furthermore, HBx can induce p53 translocation to mitochondria. HBx was also shown to influence mitochondrial dynamics through its interaction with Drp1 and Mnf2. Polymerase (Pol) contains an amino-terminal MTS that determines its mitochondrial targeting; it impact on mitochondria remains to be understood. HCV (orange): Core increases mitochondrial respiration, ROS generation, and uptake of Ca^2+^, which sensitizes PTP opening. Core also inhibits translocation of Parkin to mitochondria, favors/facilitates/promotes Bax-Bak oligomerization, and interacts with the matrix chaperone HSP60. HPV (blue): The E2 protein interacts with IMM proteins and induce expression of the matrix protein PDK1 (pyruvate dehydrogenase kinase 1); E2 also increases ROS generation in mitochondria. The E1^∧^E4 protein causes loss of ΔΨ_m_. OMM, outer mitochondrial membrane; IMM, inner mitochondrial membrane; IMS, inter-membrane space; ROS, reactive oxygen species; MTS, mitochondrial targeting sequence; ETC, electron transport chain; PTP, permeability transition pore; not determined.

Two HPV proteins, E1^∧^E4 and E2, partially localize to mitochondria (Table 1, Figure 2). E1^∧^E4 is produced by a spliced mRNA that joins the first few codons from the E1 ORF in frame to the E4 ORF, resulting in a fusion protein of about 90-120 amino acids, depending on the genotype. E1^∧^E4 is the most abundantly expressed viral protein in the mid-upper epithelial layers of productively infected lesions (reviewed by Doorbar, 2013). Doorbar et al. (1991) showed that E1^∧^E4 binds to and collapses the cytokeratin network in mature human keratinocytes. Collapse of the cytokeratin network leads to accumulation of E1^∧^E4 in mitochondria, mediated by an N-terminal leucine-rich sequence (Raj et al., 2004). E1^∧^E4-containing mitochondria dissociate from microtubules, are clustered in the perinuclear region and exhibit a severe reduction in ΔΨ_m_; these changes are associated with increased apoptosis (Raj et al., 2004).

### E2

E2 is a protein of about 365 amino acids (HPV genotype 16; its length varies among genotypes) whose expression gradually increases from the mid- to upper layers of infected lesions. This pattern of expression reflects the key role of E2 in the coordination of late events of viral replication through its influence on transcription, viral genome replication and partitioning in dividing cells, and RNA processing (reviewed by Graham, 2016).

A comparison of the properties of E2 from low-risk (genotypes 6, 11) and high-risk (genotypes 16, 18) viruses revealed stable nuclear compartmentalization of low-risk E2, and shuttling of high-risk E2 between the nucleus and cytoplasm, a property that was connected to the ability of high-risk E2 to induce apoptosis (Blachon et al., 2005). A more recent analysis of E2 trafficking provided evidence that the cytoplasmic fraction of E2 eventually accumulates in mitochondria. Lai et al. studied GFP-tagged E2 proteins from HPV-18 and HPV-6 (high- and low-risk genotypes, respectively) in a keratinocyte cell line (Lai et al., 2013). Time-lapse studies showed that HPV-18 GFP-E2 was gradually relocalized from the nucleus to mitochondria, whereas HPV-6 GFP-E2 remained predominantly nuclear and was rerouted to mitochondria only when expressed at high levels. Mass spectrometry analysis of proteins co-immunoprecipitated with HPV-18 E2 revealed the association of E2 with many mitochondrial proteins, mostly of the IMM, including subunits of Complexes III, IV, and V. Cells expressing HPV-18 GFP-E2 exhibited perinuclear clustering of the mitochondria and loss of the cristae structure. HPV-18 GFP-E2-expressing keratinocytes showed higher levels of mitochondrial ROS however, this effect did not induce cytochrome *c* release or apoptosis.

Lai et al. (2013) also showed that HPV-18 E2 stabilizes HIF-1α and induces the expression of known HIF-1α target genes, including PDK1 (pyruvate dehydrogenase kinase 1) and CAIX (carbonic anhydrase IX). These changes were accompanied by a modest but statistically significant increase in lactate production, an indication of a shift toward glycolytic metabolism. These effects were not observed in cells expressing low-risk HPV-6 E2. Although mitochondrial localization of HPV-18 E2 was evident Lai et al.'s transfection assays, their results of immunohistochemistry assays revealed cytoplasmic staining of HPV-18 E2 in a CINII lesion (grade II cervical intraepithelial neoplasia) and mainly nuclear accumulation of HPV-6 E2 in a benign condyloma.

A study by Chen et al. (2014) indicated that HPV-induced mitochondrial alterations also involve interplay between E2 and C1QBP (complement C1q-binding protein, also named gC1qR, receptor of the globular heads of complement C1q), a predominantly mitochondrial protein that has many roles, including regulation of mitochondrial and ER morphology and cell metabolism (Hu et al., 2013). Both HPV-16 E2 and C1QBP were less abundant in cervical carcinoma samples compared to non-neoplastic cervical tissue, suggesting a negative role for these proteins in the growth of cervical epithelial cells (Chen et al., 2014). Ectopic expression of E2 in keratinocytes led to upregulation of C1QBP and signs of mitochondrial dysfunction, including increased cellular ROS, augmented levels of cytosolic Ca^2+^, loss of ΔΨ_m_, and increased apoptosis; similar effects were observed in cells overexpressing C1QBP. These properties described for E2 contrast with previous observations made for the HPV oncoproteins E6 and E7, which downregulate C1QBP expression and protect cells from apoptosis (Gao et al., 2011; Chen et al., 2014). It is noteworthy that HCV Core protein also binds to C1QBP, resulting in impaired cytokine production (Song et al., 2016).

## Concluding remarks

Mitochondria play a central role in key biological processes, including energy conservation, cell death and Ca^2+^ signaling. These processes are involved both in the control of physiological tissue homeostasis and in the process of neoplastic transformation. It is thus not surprising that tumor viruses have developed common strategies to impinge on mitochondrial function. The experimental evidence collected so far suggests five major mechanisms by which proteins coded by tumor viruses interact with mitochondria (see Figure 2): (i) direct inhibition of apoptosis by viral Bcl-2 homologs; (ii) deregulation of cellular bioenergetics through the interaction with ETC components and inner mitochondrial membrane complexes; (iii) changes in the mitochondrial production of ROS, which indirectly influence cell turnover; (iv) changes in mitochondrial Ca^2+^ uptake, which influence Ca^2+^ homeostasis and Ca^2+^-dependent signal transduction pathways; (v) interaction with OMM proteins that control innate immunity. Mitochondrial viral proteins can also alter mitochondrial morphology through interactions with the fission/fusion machinery.

Although much experimental information supports these points of interaction between tumor virus-encoded proteins and mitochondria, it must be emphasized that in most cases these results were obtained from experimental systems based on the ectopic overexpression of the viral protein of interest, in the absence of other viral proteins, and in cell types that are not the targets of natural infection by the viruses, conditions that may not recapitulate the situation in naturally infected cells. Further investigation is thus needed in order to validate and clarify the effects of viral-encoded mitochondrial proteins in the context of the viruses' life cycle and in connection to their pathogenic properties in the natural host.

In addition to providing information about the life cycle and pathogenesis of human tumor viruses, the examination of virus-mitochondrial interactions using more refined experimental systems will enhance our understanding of basic cellular processes controlled by mitochondria, thus paving the road to the identification of new strategies to clear viral infections and treat cancer patients.

## Author contributions

IC prepared the paragraph on HPV and Table 1. GS prepared the paragraph on EBV and Figures 1, 2. MS-B prepared the paragraph on HSHV. VR prepared the paragraph on HTLV-1. VC prepared the paragraph on HBV and revised the final version of the paper. DD prepared the paragraph on HCV, Table 2 and revised the final version of the paper.

### Conflict of interest statement

The authors declare that the research was conducted in the absence of any commercial or financial relationships that could be construed as a potential conflict of interest.
